# The Perspective of mHealth in the Self-Assessment of the Parkinson’s Disease. Comment on Kalafati et al. Testing of Motor Coordination in Degenerative Neurological Diseases. *Healthcare* 2022, *10*, 1948

**DOI:** 10.3390/healthcare11060850

**Published:** 2023-03-14

**Authors:** Daniele Giansanti

**Affiliations:** Centre Tisp, The Ialian National Institute of Health, 00161 Rome, Italy; daniele.giansanti@iss.it; Tel.: +39-06-49902701

Regarding the *research article* “*Maria Kalafati, Athanasios Kakarountas and Elisabeth Chroni*, Testing of Motor Coordination in Degenerative Neurological Diseases”, published in *Healthcare* [1], I found that this is a stimulating work in the field of Biomedical Engineering with interesting clinical perspectives.

As you highlighted in the study [1]:

(1) Parkinson’s disease (a progressive movement disorder caused by the death of dopamine-producing cells in the midbrain) is the most prevalent movement disorder of the central nervous system and affects more than 6.3 million people in the world;
(2) Changes in the motor functions of patients are not easy to be clearly observed on time by the clinicians and to make the most well-informed decisions for the treatment;
(3) It is important in light of points (1, 2) to develop bioengineering methods integrated with modern mobile technologies, capable of (a) being easily used by patients with Parkinson’s Disease (PD) and (b) providing useful parameters to allow decisions based on quantitative data within the PD.

Your study [1] details the designing, developing, and evaluating of a mobile app using a pressure pen, which collects quantitative and objective information about PD patients, thus allowing clinicians to understand better and make assumptions about the severity and the stage of Parkinson’s disease. The app described by you allows the execution of a dynamic spiral test through a pen pressure and obtaining a series of important parameters such as Spiral Deviation, Total Time, and Pen Pressure through a computerized system. The study reports the potential of the system as a telemonitoring method within the PD to remotely perform screening tests and maintain the history of all the patient’s measurements.

I believe it is very important to introduce app-based systems and, more generally, *mHealth* solutions that allow the *health domain* to provide quantitative data on a pathology as important as Parkinson’s disease. I also believe that these systems are, so to speak, also pioneers based on the introduction of wearable tools for *mHealth-based self-assessment* for PD. Moreover, this is very motivating because *mHealth* seemed to be more concentrated only on specific diseases such as diabetes, heart, and lung diseases [2,3,4].

When the Special Issue “Assistive Technologies, Robotics, and Automated Machines in the Health Domain” https://www.mdpi.com/journal/healthcare/special_issues/Assistive_Technologies_Robotics_Automated_Machines_Health_Domain (accessed on 5 March 2023) [5] was launched, one of the objectives [6] was to give scholars the opportunity to broaden the boundaries of studies in this area.

*mHealth* is recently increasingly arousing the interest of scholars in various sectors of the *health domain*. A brief search on Pubmed [7] with the search key (*mobile health [Title/Abstract]) AND (self assessment [Title/Abstract]*) shows reviews of *a growing attraction under different and new perspectives* [8,9,10,11,12].

The study by Alanzi [11] revealed that various applications were developed during the COVID-19 pandemic, also available in Google Play, for different functions such as contact tracing, awareness building, appointment booking, online consultation, etc. However, only a few applications have integrated various functions and features such as self-assessment, consultation, support and access to information.

The study by Claessens et al. [10] reported that numerous digital tools to self-assess visual acuity were introduced and concluded that these have the potential to increase access to eye care, also considering it is expected that the accuracy of the current tools will improve with every iteration in technology development.

Bonnechere et al. [9] highlighted that mHealth is a promising tool for the self-assessment and rehabilitation of people with multiple sclerosis, even if more studies and works are needed to put these solutions in stable routine applications.

Ni et al. [8] faced the use of mHealth for self-assessment in lung cancer. They showed that patients with lung cancer have diversified supportive care needs after discharge. A bottom-up and stepwise approach to developing mobile health-based self-management support tools has been demonstrated to be feasible and valuable.

Santo and Redfern [12] described how recent research has shown that mHealth apps can be beneficial in terms of improving hypertension self-assessment, treatment, and control, being especially useful to help differentiate and manage true and pseudo-resistant hypertension.

I am convinced that it could be useful and stimulating *to enlarge the concept of self-assessment also in PD.* In the past, before the smartphone boom, I was interested in using wearable methods to obtain quantitative parameters in PD.

Together with other coauthors, we developed a specific pedometer for patients with Parkinson’s disease, the parameters connected with the path being strategic and also for the diagnostic monitoring of this pathology [13].

Subsequently, other authors and I developed a system for personal computers for monitoring the activity on the video terminal [14], which made it possible to record the trajectories of the movement of the mouse even in the execution of tasks. Nevertheless, with great scientific honesty, I must remember that we never had the idea of using it in PD [8].

I believe that *mHealth* today allows, as you have also highlighted [1], new opportunities in PD monitoring/assessment.

Based on what I wrote above, I would like to open a discussion on this topic with this comment with you or with other authors interested in the discussion.

In particular, I would like to have an opinion on how you or other authors see the development of *mHealth* self-assessment technologies on PD.

I would, therefore, very much appreciate a *reply* in this regard.
